# Case Report: Pediatric alloplastic nose reconstruction with a 3D printed patient specific titanium implant

**DOI:** 10.3389/fsurg.2024.1330889

**Published:** 2024-03-13

**Authors:** Matthias Ureel, Daniel Dadjam, Nicolas Dhooghe, Maarten De Jong, Renaat Coopman

**Affiliations:** ^1^ Department of Plastic and Reconstructive Surgery, Division of Oral and Maxillofacial Surgery, Ghent University Hospital, Ghent, Belgium; ^2^ Department of Biomedical Engineering, Swiss MAM Research Group, University of Basel, Allschwil, Switzerland; ^3^ Anaplastologist, Maastricht University Medical Center+, Maastricht, Netherlands

**Keywords:** nose reconstruction, pediatric, patient specific implant, prosthesis, dog bite

## Abstract

An 11-year-old girl presented at the emergency service with a nasal defect caused by a dog bite in the midface. Autologous nose reconstruction in the pediatric population is challenging due to donor site morbidity and remaining facial growth. Temporary prosthetic treatment is difficult to accept due to problems with retention. We present an innovative solution using a 3D printed patient specific titanium implant for support of a nasal prosthesis. With preoperative 3-dimensional planning, the implant can be designed to find fixation in the areas with the best bone quality, avoid potential damage to tooth buds and dental roots and avoid interference to soft tissues such as the nasal septum. Clear communication between the anaplastologist, surgeon and medical engineer is crucial for treatment success. The impact of facial growth is still unclear and will have to be assessed.

## 1 Introduction

Nasal defects can be caused by oncologic resections, congenital deformities, or traumatic injuries. In the adult population, there are several autologous and prosthetic reconstructive options which have been well described in literature (1–3). In the pediatric population on the other hand, treatment options are limited due to considerations of the remaining facial growth and donor site morbidity (4–6). Autologous reconstruction is complex, requires multiple surgeries and necessitates special expertise. More so, the donor site has significant morbidity, and the long treatment time can result in an important psychosocial burden (4, 7). Prosthetic treatment with adhesives results in a shorter treatment time and fast return to society and social life. However, this type of treatment is less accepted due to loosening of the prosthesis during activities, potential allergic reactions, and frequent renewals due to wear (2, 8, 9). Endosseous fixation for retention of the prosthesis has resolved many of the issues encountered with adhesive retained prosthesis (2, 8, 10) but has rarely been used in the pediatric population due to the presence of teeth buds, dental roots, and the remaining facial growth. The report adheres to the tenets of the Declaration of Helsinki. Written informed consent of the patient was obtained.

## 2 Case presentation

An 11-year-old female patient in good general health was admitted to the emergency service of a level I trauma center with an avulsed nose and extensive lacerations on the upper left arm after a canine attack. She had no known allergies, no relevant medical history and was fully vaccinated as recommended by the health care authorities.

**Figure 1:**
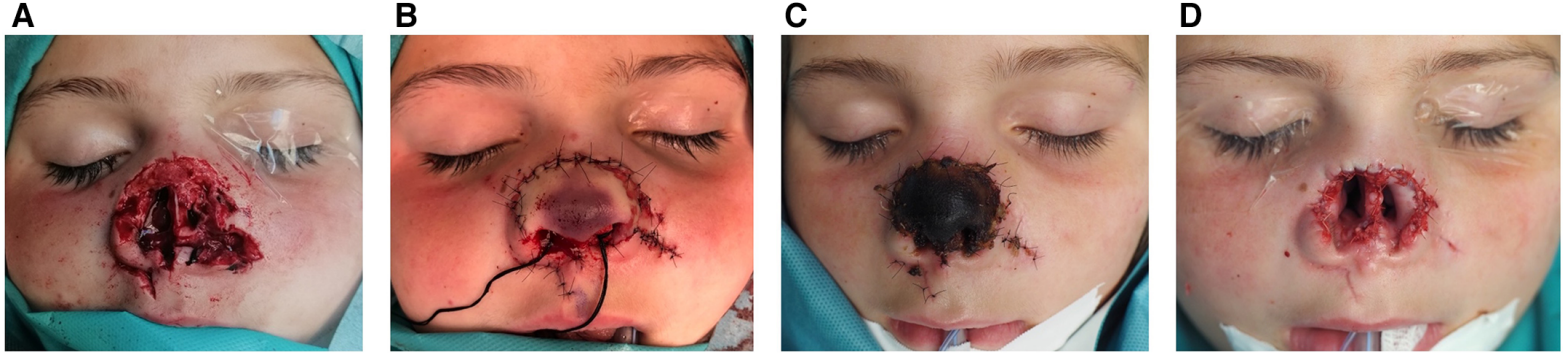
(**A**) Initial presentation after traumatic nasal avulsion; (**B**) immediate microvascular reattachment of the avulsed tissue; (**C**) necrosis of the avulsed tissue, 1 week after anastomosis; (**D**) situation after removal of the necrotized tissue.

At the emergency service, intravenous antibiotic prophylaxis with 1 g amoxicillin, 100 mg clavulanic acid and 800 mg paracetamol was administered. She was immediately transferred to the operating room to debride and cleanse the wound. An attempt was made to reattach and revascularize the avulsed tissue through vascular microanastomosis at the columella (columellar branches of the superior labial artery of the facial artery). Unfortunately, no vessels of sufficient quality could be found on the avulsed segment, due to the small size and the tearing nature of the trauma. Multiple attempts to perform an arterial microvascular anastomosis did not show any perfusion. The segment was reattached as a composite graft and covered with a protective dressing. Eleven days later, the tissue necrotized and had to be removed (Figure 1).

Two weeks after the incident, the reconstructive options were discussed with the patient and her parents. Autologous reconstruction was denied due to donor site morbidity, long treatment time and unpredictable long-term results. To avoid damage to the tooth buds an adhesive retained nasal prosthesis was preferred over endosseous fixation with implants. Albeit the aesthetic result was excellent, the prosthesis loosened during sport and social activities. It was then decided to search for osseous fixation by using a patient specific implant (PSI).

### 2.1 Following aspects were considered during the design of the implant

- Preserve as many of the remaining soft tissue as possible, to allow the possibility for an autologous reconstruction after completion of facial growth.
- Avoid tooth buds and dental roots, especially the upper canines that are positioned paranasal at this age. Conventional endosseous implants in the nasal floor could damage the dental apices.
- The bone supported Epi-plate system finds paranasal support and can be bent around the septum. However, the septum is prominent in this case leaving less space for the prosthesis and complicating cleaning of the support structure.
- We assumed that four fixation screws on each side would be sufficient to achieve long-term stability.
- Prevent rotational movements of the prosthesis and assure only one possible prosthetic position. Therefore, three 2 mm diameter round bars were designed for fixation. Priority was given to clip attachments instead of magnets because of their compact size.
- Provide enough prosthetic space to facilitate the creation of an aesthetic prosthesis.
- Facilitate easy cleaning of the PSI.

**Figure 2:**
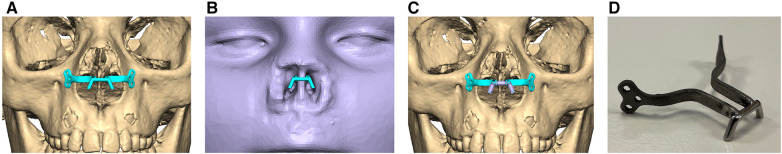
(**A**) Virtual planning of the patient specific implant in frontal view. (**B**) Position of the PSI with surrounding soft tissue mask in frontal view. (**C**) PSI with fitted retention clips. (**D**) 3D printed titanium patient specific implant (CADskills bv, Gent, Belgium).

During this phase, communication between the anaplastologist, surgeon and medical engineer is essential for a good outcome. Aside from the important surgical aspects, the position of the PSI is crucial for the final prosthetic result (Figure 2).

### 2.2 Surgical placement of the PSI

Approximately 1 year after the trauma, the patient specific implant was placed under general anesthesia. The original scar on the transition of nasal mucosa and skin was used for surgical access. After careful dissection the periosteum was incised, and the paranasal bone visualized. Approximately two millimeters of septal cartilage had to be removed to achieve perfect seating of the PSI. The PSI was fixed with 4 self-tapping screws of 1.5 × 5.0 mm on each side (KLS Martin, Tuttlingen, Germany). The mucosa was closed with resorbable interrupted sutures and the skin with nylon interrupted sutures. Post-operative wound care consisted of daily application of antibiotic ointment on the surgical site, oral antibiotic prophylaxis with amoxicillin clavulanic acid 1 g/100 mg 4 times daily for 24 h, and pain control with weight adjusted paracetamol and ibuprofen.

### 2.3 Postoperative follow-up and evaluation of the result

**Figure 3:**
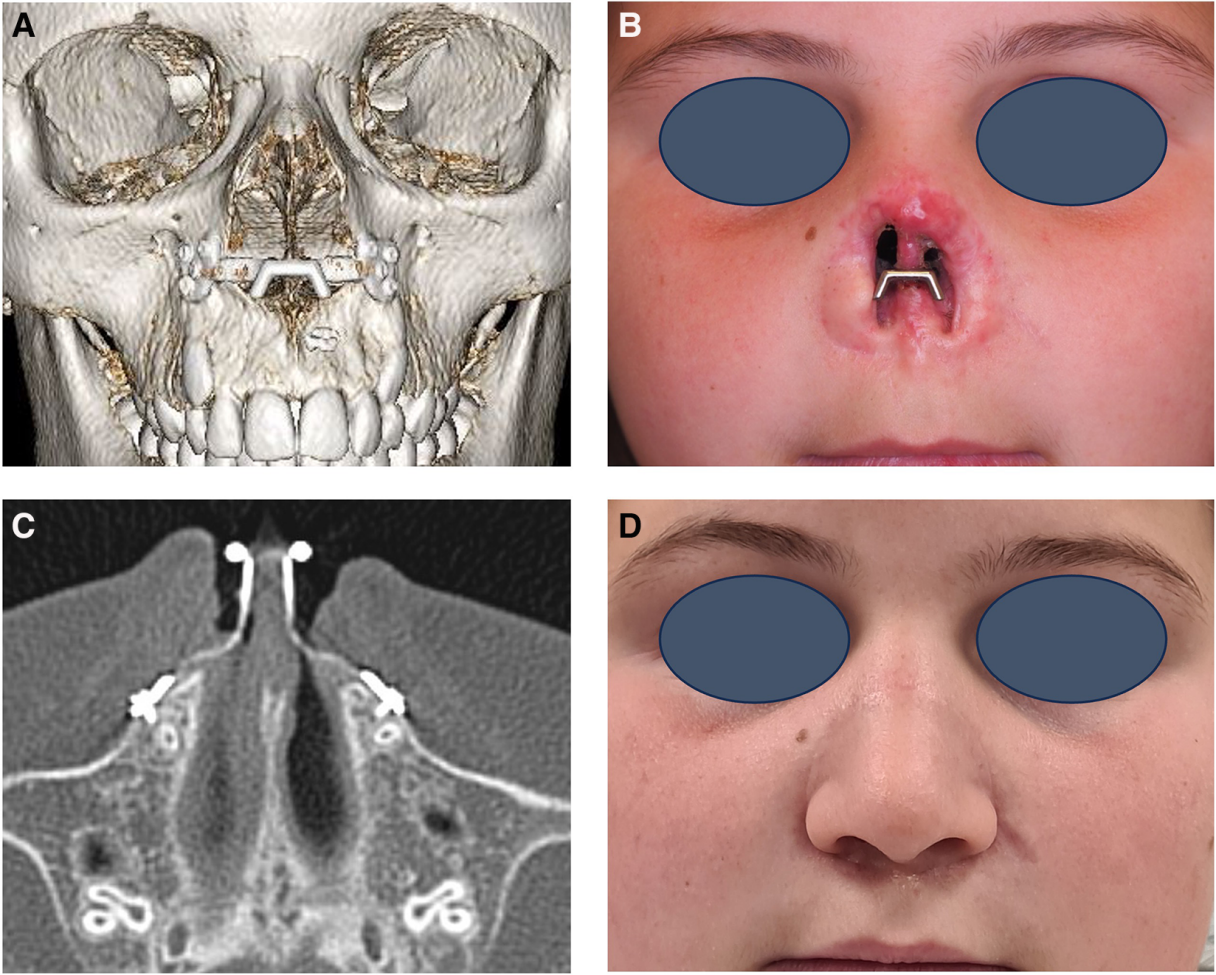
Follow-up 5 months postoperative. (**A**) 3D reconstruction of the postoperative computed tomography (CT) scan with the patient specific implant (PSI) in place. (**B**) Clinical picture of the patient with the PSI in place. (**C**) Axial view of the postoperative CT-scan indicating a close position of the osteosynthesis screws to the root of the canine teeth. (**D**) Clinical picture of the patient with the nasal prosthesis fixed to the PSI.

**Figure 4:**
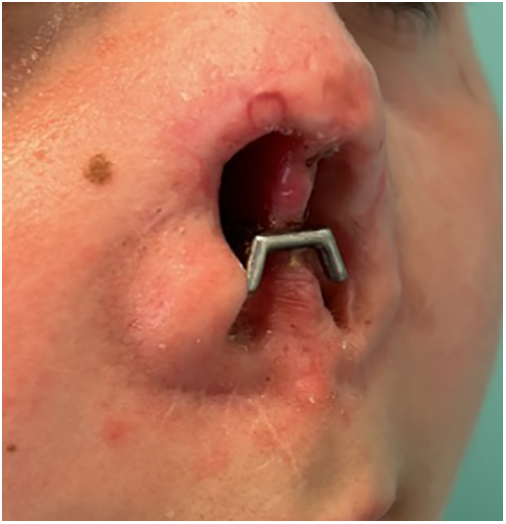
Follow-up 18 months postoperative. Clinical picture demonstrating growth of the nasal septum towards the PSI.

Post-operative computed tomography imaging showed a good seating of the patient specific implant on the paranasal bone without damage to the dental roots (Figure 3). Compared to the preoperative planning the implant was placed too low. Two months after surgery the prosthesis could be fixed on the PSI with good retention and aesthetics. The patient has had no complaints and no signs of infection or irritation. Follow-up for 18 months has been uneventful, however we now see contact of the nasal septum with the PSI (Figure 4).

## 3 Discussion

Traumatic nasal defects in the pediatric population are difficult to treat. In the acute setting the wounds should be cleaned and debrided (11–13). Antibiotics, rabies and tetanus prophylaxis need to be administered if the vaccination status of the animal is unknown or is unable to be observed (14–16). If the amputated nose is present and in good shape, microvascular reattachment should be attempted (17, 18). Bite traumas present several reconstructive difficulties, due to soft tissue tearing, crush trauma and risk of infection (11, 18–20).

Unfortunately, in this case the reattached tissue necrotized and had to be removed. In the adult population, autologous reconstructive options for nasal avulsion have been extensively documented in the literature, such as the commonly used paramedian forehead flap or the auricular flap (21). Nasal reconstruction in children is scarcely documented. It is advised to perform the surgery as in the adult population, preferably after the age of 10. Corrective surgeries at a later age are often necessary (22). Another alternative is the Washio retroauricular flap as the donor site is hidden and popular flaps such as the paramedian forehead flap can still be used at a later stage (23). This technique requires a two-stage approach. Autologous reconstruction in children is due to the inability of own tissue to age and evolve with the patient unpredictable. Combined with the morbidity of the donor site and the necessity of two surgeries, this solution was not preferred by the parents (24).

The evaluation of children as potential candidates for VCA for facial reconstruction has already been commented by many leaders in VCA, psychologists and psychiatrists, ethicists, and researchers. There are many barriers precluding inclusion of pediatric patients as potential candidates: the difficulty of establishing at which age a child might be aware of the procedure implications, parents' right to give consent, psychological issues and whether VCA is worth the tradeoff for a better, but shorter life as they would face the complications associated with life-long immunosuppression (25).

Nasal reconstruction with an adhesive prosthesis is a viable and elegant option as it provides a fast and esthetically pleasing solution. It can be perceived as a temporary solution before definite autologous reconstruction is performed. It does however not provide comfort during function as the prosthesis loosens easily. More so, allergic reactions on the adhesives have been reported. In this case the adhesive retained prosthesis was aesthetically pleasing but was not accepted by the child as it loosened during social activities.

Endosseous implant retained nasal prosthesis can overcome the above-mentioned disadvantages, but the classic implant positions in the nasal floor could damage the tooth buds. Implantation in the glabella would result in a new scar and tissue loss, and would also result in less prosthetic space hindering the creation of an elegant prosthesis.

To circumvent the limitations stated above, a patient specific titanium implant was designed, and 3D printed. During this phase, communication between the anaplastologist, surgeon and medical engineer is essential for a successful result. There needs to be enough fixation in the bone to withstand the forces of the prosthesis, thereby avoiding any interference with the tooth buds and the nasal septum. Prosthetically there needs to be enough space to create the nasal prosthesis and to be able to clean the implant. And for the patient it should be easy to place and remove the prosthesis and clean the peri-implant tissue.

During surgery it was difficult to assess the correct position of the implant because the contact area of the feet of the PSI and the bone is limited. The implant was placed too low compared to our preoperative planning. We suggest using a surgical guide or intraoperative navigation to position the implant correctly. During surgery 2 mm of septal cartilage had to be removed. Possibly the interference was caused by the lower position of the implant or growth of the septum as the preoperative CT scan was made 12 months before the day of the surgery.

Eighteen months after surgery, the patient reports no signs of infection or pain after surgery. She finds it easy to remove and place the prosthesis and sometimes even forgets to wear it. Cleaning poses a challenge, especially behind the arms emerging from the paranasal skin. Retention of the prosthesis is excellent during social activities and sports. Overall, her experience reflects a positive adaptation to the prosthesis.

This eleven-year-old patient is not yet fully grown. As described in literature the remaining growth of the female midface at that age is expected to be around 9%. The growth of the female nasal septum and nose is expected to be completed at 16 years of age (26, 27). During follow-up the nasal septum has grown towards and partially around the titanium implant. We assume that the prosthesis will block further growth of the nasal septum. However as soon as the patient complains of tissue interference or problems with prosthesis positioning it might be necessary to remove part of the nasal septum or create a new patient specific implant. The prosthesis will need to be adjusted conform her age. Frequent follow-up appointments are necessary to further assess any interference of the PSI with the soft tissues and to assist in cleaning the interface of the soft tissue and the PSI. When the patient is outgrown, autologous reconstruction will still be possible as the implant can easily be removed without sacrificing soft tissue.

An important disadvantage of this treatment option is the financial impact for the patient. The health insurance in Belgium does not reimburse the PSI.

## Data Availability

The original contributions presented in the study are included in the article/Supplementary Material, further inquiries can be directed to the corresponding author.
